# Supplementary material for: Analysis of PPARγ Signaling Activity in Psoriasis
Source: Int J Mol Sci. 2021 Aug 10;22(16):8603. doi: 10.3390/ijms22168603 (PMC8395241; doi:10.3390/ijms22168603)
Supplement: Supplementary file 1 [file ijms-22-08603-s001.zip › Supplemental materials_Analysis of PPARg signaling activity in psoriasis/Pathway models/Models images and html files/PPARG inhibits phenotypes assotiated with psoriasis/30364.html]

coronary atherosclerosis


# Disease coronary atherosclerosis

|  |  |
| --- | --- |
| URN | urn:agi-meshdis:Coronary%20Arteriosclerosis |
| Total Entities | 0 |
| Connectivity | 1519 |
| Name | coronary atherosclerosis |

---

|  |  |
| --- | --- |
| Pathway | PPARG inhibits phenotypes assotiated with psoriasis |

---

|  |  |
| --- | --- |
| MedScan ID | 9205188 |

---

|  |  |
| --- | --- |
| Alias | coronary arterial atheroscleroses |
|  | coronary sclerosis |
|  | coronary artery atheroscleroses |
|  | atherosclerotic cardiovascular disease |
|  | arterosclerotic cardiac diseases |
|  | atherosclerotic heart diseases |
|  | arteriosclerotic heart diseases |
|  | arteriosclerotic cardiac diseases |
|  | arteriosclerosis of the coronary artery |
|  | arteriosclerotic cardiovascular diseases |
|  | coronary arterial atherosclerosis |
|  | arteriosclerotic coronary heart disease |
|  | coronary artery atherosclerosis |
|  | atheriosclerotic cardio-vascular disease |
|  | atheriosclerotic coronary heart diseases |
|  | atherosclerotic cardiovascular diseases |
|  | atherosclerotic cardio-vascular disease |
|  | coronary arterial arteriosclerosis |
|  | arteriosclerotic cardio-vascular disease |
|  | coronary arterial sclerosis |
|  | arteriosclerotic heart disease |
|  | atherosclerotic cardio-vascular diseases |
|  | coronary artery sclerosis |
|  | arteriosclerotic cardiac disease |
|  | atheriosclerotic cardio-vascular diseases |
|  | atheriosclerotic cardiovascular diseases |
|  | atherosclerosis of the coronary arteries |
|  | atherosclerotic coronary vascular diseases |
|  | arteriosclerotic coronary heart diseases |
|  | atherosclerotic coronary heart diseases |
|  | arterosclerotic cardiac disease |
|  | arteriosclerosis of the coronary arteries |
|  | arteriosclerotic cardiovascular disease |
|  | atherosclerosis of the coronary artery |
|  | atherosclerotic coronary vascular disease |
|  | coronary atherosclerosis |
|  | atherosclerotic coronary heart disease |
|  | atheriosclerotic coronary heart disease |
|  | ASCVD |
|  | coronary artery arteriosclerosis |
|  | atheriosclerotic cardiovascular disease |
|  | arteriosclerotic cardio-vascular diseases |
|  | atherosclerotic heart disease |
|  | coronary arteriosclerosis |
|  | Arteriosclerosis, Coronary |
|  | Atherosclerosis, Coronary |
|  | Arteriosclerosis Coronary Arteries |
|  | Arteriosclerosis Coronary Artery |

---

|  |  |
| --- | --- |
| MeSH ID | M0005188 |

---

|  |  |
| --- | --- |
| MeSH Heading | Coronary Arteriosclerosis |

---
